# Stroma Regulates Increased Epithelial Lateral Cell Adhesion in 3D Culture: A Role for Actin/Cadherin Dynamics

**DOI:** 10.1371/journal.pone.0018796

**Published:** 2011-04-18

**Authors:** Karen F. Chambers, Joanna F. Pearson, Naveed Aziz, Peter O'Toole, David Garrod, Shona H. Lang

**Affiliations:** 1 YCR Cancer Research Unit, Department of Biology, University of York, Heslington, York, United Kingdom; 2 Genomics Lab, Technology Facility, Department of Biology, University of York, Heslington, York, United Kingdom; 3 Imaging and Cytometry Lab, Technology Facility, Department of Biology, University of York, Heslington, York, United Kingdom; 4 Faculty of Life Sciences, University of Manchester, Manchester, United Kingdom; 5 King Saud University, Riyadh, Saudi Arabia; University of California, Merced, United States of America

## Abstract

**Background:**

Cell shape and tissue architecture are controlled by changes to junctional proteins and the cytoskeleton. How tissues control the dynamics of adhesion and cytoskeletal tension is unclear. We have studied epithelial tissue architecture using 3D culture models and found that adult primary prostate epithelial cells grow into hollow acinus-like spheroids. Importantly, when co-cultured with stroma the epithelia show increased lateral cell adhesions. To investigate this mechanism further we aimed to: identify a cell line model to allow repeatable and robust experiments; determine whether or not epithelial adhesion molecules were affected by stromal culture; and determine which stromal signalling molecules may influence cell adhesion in 3D epithelial cell cultures.

**Methodology/Principal Findings:**

The prostate cell line, BPH-1, showed increased lateral cell adhesion in response to stroma, when grown as 3D spheroids. Electron microscopy showed that 9.4% of lateral membranes were within 20 nm of each other and that this increased to 54% in the presence of stroma, after 7 days in culture. Stromal signalling did not influence E-cadherin or desmosome RNA or protein expression, but increased E-cadherin/actin co-localisation on the basolateral membranes, and decreased paracellular permeability. Microarray analysis identified several growth factors and pathways that were differentially expressed in stroma in response to 3D epithelial culture. The upregulated growth factors TGFβ2, CXCL12 and FGF10 were selected for further analysis because of previous associations with morphology. Small molecule inhibition of TGFβ2 signalling but not of CXCL12 and FGF10 signalling led to a decrease in actin and E-cadherin co-localisation and increased paracellular permeability.

**Conclusions/Significance:**

In 3D culture models, paracrine stromal signals increase epithelial cell adhesion via adhesion/cytoskeleton interactions and TGFβ2-dependent mechanisms may play a key role. These findings indicate a role for stroma in maintaining adult epithelial tissue morphology and integrity.

## Introduction

Cell shape is controlled by environmental, chemical and mechanical signals provided by extracellular matrix, growth factors, the cytoskeleton and cell adhesion molecules. The ability of a cell to change shape is important during embryonic morphogenesis and the functional development of adult tissue architecture [1]. During normal development, epithelia remodel extensively indicating that their adhesions are capable of plasticity. During the development of diseases such as cancer the breakdown of cellular morphology is attributed to the aberrant expression of adhesion molecules. Therefore a better understanding of the regulation of molecules involved in cellular morphology and adhesion is essential to increase our understanding of epithelial morphogenesis and disease. Most commonly, epithelial cell morphology is studied in tissue culture on solid substrata or in developing embryos. Alternative models are required to understand morphology and function in adult human tissue and to support research into human disease. To this end 3D culture models are beginning to provide useful tools with which to study adult epithelial tissues, since they reflect tissue architecture and function [2], [3].

Previously we demonstrated, using human primary cell cultures, that co-culturing stroma with epithelial cells grown as 3D spheroids increased lateral epithelial cell adhesion [4], whilst in monolayer stroma causes scattering of epithelial cells [5]. Ultrastructural analysis indicated that the presence of stroma increases the numbers of desmosomes and other lateral adhesions. E-cadherin and the desmosomal cadherins are widely studied intercellular adhesion molecules, which have important roles in morphogenesis, development and tissue patterning [6], [7]. E-cadherin forms homophilic adhesions and links to the actin cytoskeleton at adherens junctions and tight junctions. E-cadherin-mediated adhesion can increase the area of surface contact between cells, leading to changes in cell shape from spherical to polygonal [8]. Cell shape is also influenced by the mechanical tension created by the actin cytoskeleton. Adhesive and cortical tensions are not independent but are dynamically regulated through the interaction of E-cadherin and the actin cytoskeleton [9]. In contrast, desmosomes form strong adhesions that are linked to the intermediate filament system and are essential for tissue integrity [10], [11]. Their adhesion molecules are the desmosomal cadherins desmoglein (Dsg) and desmocollin (Dsc). Desmosomes and adherens junctions are mutually dependent [12].

The stromal signalling molecules that control cell adhesion remain poorly defined. In adult prostate, stromal cells provide connective tissue surrounding the epithelium; they express the androgen receptor and produce important signals to control the maintenance and differentiation of the epithelial population [13]. Primary stromal cultures are a mix of fibroblasts and smooth muscle cells, which can induce a more complete differentiation of epithelial cells in 3D and monolayer [4], [14]. However there are several candidate molecules for stromal–epithelial signalling during development. HGF is recognized as a stromal mediator of prostate epithelial growth and motility [15]. Likewise, FGF7 and FGF10 have been implicated in androgen induced ductal growth and branching morphogenesis during embryogenesis [16], [17]. In addition, members of the TGFβ superfamily (TGFβ, bone morphogenic proteins and activins) have roles in mouse prostate epithelial morphogenesis [18], [19], [20]. Conversely, paracrine signalling by TGFβ and CXCL12/SDF-1 have also been implicated in prostate tumour progression [21].

To determine which stromal signalling molecules control epithelial cell adhesions in 3D primary cell culture we looked for changes to the expression of desmosomal proteins and E-cadherin and carried out microarray analysis. Our results indicated that stroma did not affect the overall levels of E-cadherin or desmosome expression, but increased the co-localisation of E-cadherin with actin at basolateral junctions. Several signalling pathways were significant during growth of epithelial cells co-cultured with stroma in Matrigel. Through the use of small molecule inhibitors, TGF-β signalling was shown to play a vital role in the regulation of E-cadherin and F-actin co-localisation and epithelial paracellular permeability.

## Results

### Co-culture of epithelia with stroma increases lateral epithelial cell adhesion in 3D cultures

**Figure 1:**
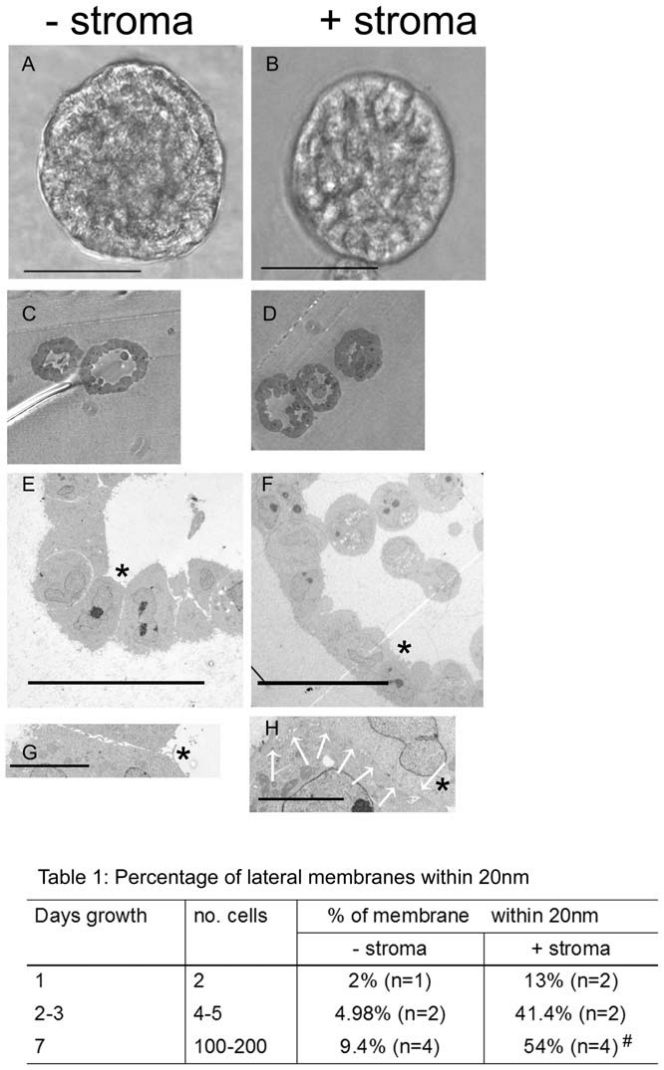
Stromal co-culture increases lateral epithelial cell adhesion. (A and B). Phase images of 3D BPH-1 acini grown with (B) and without stroma (A) for 8 days, bars  = 50 µm. Thick sections (C,D) and TEM (E,F) of mid-sections through BPH-1 acini, grown with (D,F) and without stroma (C,E). Bar  = 20 (E) or 50 µm (F). (G and H). High magnification TEM images of the junctions marked by the asterisks in (E) and (F). The lateral cell-cell contact shown in (H) is highlighted by white arrows, bars  = 5 µm. Images are representative of three spheroids analysed per experiment. The values in table 1 have standard deviations of ±15%, #  = p<0.018.

Our preliminary studies have shown that primary epithelial prostatic cells grown in Matrigel develop into acinus-like spheroids. In the presence of stroma the epithelia became more polarised and exhibited increased lateral cell adhesions [4]. To overcome the heterogeneity inherent in primary cultures, we studied the effect of stroma on 3D cultures of a prostate cell line (BPH-1). At low magnification the spheroids appeared hollow (depending on the plane of cut) but otherwise similar in the presence and absence of stroma **(** **Figure 1A–1D** **)**. However, ultrastructural analysis of acini indicated that BPH-1 cells also demonstrated increased lateral cell adhesion in response to stromal co-culture **(** **Figure 1E–1H** **)**. At high magnification, we observed that in the absence of stromal cells, the epithelial cells were loosely adherent with large intercellular spaces. Addition of stroma led to reduction of these spaces and increased cell-to-cell contacts **(** **Figure 1H** **)**. To quantify the extent of cell-cell adhesion we measured the percentage of opposing membranes that were within 20 nm of each other from electron micrographs of cells with visible nuclei **(** **Table 1** **)**. At all stages of acinus development we found that stroma increased the proximity of the lateral junctions. Stromal co-culture for 7 days significantly increased the extent of 20 nm epithelial cell-to-cell contact. A significant increase may have occurred earlier but it was difficult to perform accurate measurements for small acini (2 to 4 cells). The acini formed by BPH-1 cells closely resemble those formed by primary epithelial cells. Therefore BPH-1 cells represent a tissue-relevant and robust model for the further study of stromal signalling mechanisms.

**Table 1:** Percentage of lateral membranes within 20 nm.

Days growth	No. cells	% of membrane	within 20 nm
		−stroma	+stroma
1	2	2% (n = 1)	13% (n = 2)
2–3	4–5	4.98% (n = 2)	41.4% (n = 2)
7	100–200	9.4% (n = 4)	54% (n = 4)#

The values in table 1 have standard deviations of ±15%, #  = p<0.018.

### Stroma does not control the levels of E-cadherin and desmoglein expression

**Figure 2:**
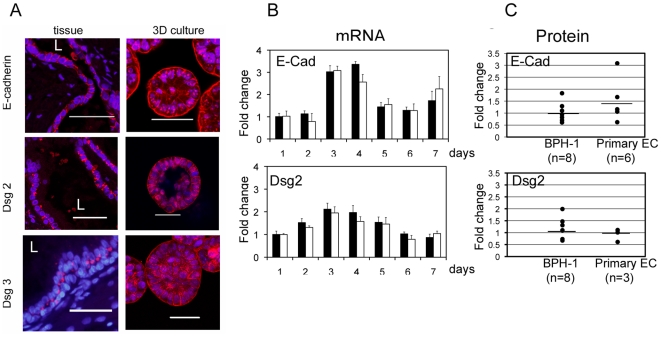
Stroma does not effect E-cadherin or Dsg2 mRNA or protein expression levels. (A). The localisation of E-cadherin and Desmogleins in human tissue and BPH-1 acini sections by immunostaining for E-cadherin, Dsg2 or Dsg3 (red) (A). Nuclei were counterstained with DAPI (blue), bars  = 50 µm, L  =  lumen. (B). TaqMan assay was performed on mRNA isolated from 3D BPH-1 acini cultured with (white bars) or without stroma (black bars), over 7 days culture shows that stroma does not increase E-Cadherin or Dsg2 mRNA expression at any time-point. The average fold difference in gene expression was expressed relative to GAPDH. Error bars represent standard deviation of three replicates and the experiment was reproduced twice. (C). Quantitative western blotting was performed on BPH-1 cells or primary epithelial cultures (primary-EC) grown in Matrigel with or without stroma. The levels of E-cadherin or Dsg2 protein expression did not significantly increase in either BPH-1 acini or primary acini cultured in the presence of stroma. Protein levels were measured by densitometric analysis and normalised to β-actin. A mean fold change in protein expression was calculated for cells grown with stroma in comparison to those without stroma. Individual experiments are represented as dots and the mean value is shown by the bar.

To discover whether stromal cells were controlling the expression of cell adhesion molecules in 3D culture we confirmed the expression and localisation of E-cadherin and desmosomal proteins in prostate tissue and 3D BPH-1 cell cultures, using semi-quantitative RNA expression and immunostaining. As expected, we detected high expression of E-cadherin, Dsg2, 3, 4 and Dsc2, 3 and low expression of Dsg1 and Dsc1 **(** **Figure 2** **and S1).** E-cadherin was expressed throughout BPH-1 acini at the basal and lateral membranes, following the same expression pattern as tissue. In tissue, Dsg2 expression was located predominantly at the lateral membranes and in acini there was additional expression at the basal membrane. Although Dsg3 expression was found in the lateral membranes of BPH-1 acini it was not found at the lateral cell membranes of tissue and was not considered further. High magnification images of acini indicated E-cadherin was associated with cellular protrusions at the basal membrane and that desmosomal staining was characteristically punctate but was not associated with protrusions **(Figure S2A and S2B)**. Using confocal microscopy we observed no differences in the level or location of E-cadherin or Dsg2 expression in BPH-1 acini grown with stroma compared to those without.

To determine whether stromal cells can control the levels of expression of E-cadherin and Dsg2 in acini we measured mRNA and protein levels over time. The expression of E-cadherin and Dsg2 mRNA peaked at 3-4 days culture in BPH-1 acini, but no significant difference was found between cultures with or without stroma **(** **Figure 2B** **)**. Membrane expression of E-cadherin was observed after 1 day in 3D culture [22], whilst Dsg2 membrane expression was not seen until day 4 **(Figure S2C)**. Quantitative analysis of E-cadherin protein in BPH-1 acini grown in the presence or absence of eight different stromal cultures showed that, on average, stroma did not significantly increase E-cadherin expression **(** **Figure 2C** **)**. Similarly, stromal cultures did not affect Dsg2 protein expression in either BPH-1 or primary epithelial acini **(** **Figure 2C** **)**. In summary, although it increases lateral cell-cell adhesion, stroma does not increase the mRNA or protein levels of E-cadherin or Dsg2 in BPH-1 acini.

### Stromal signalling increases E-cadherin and F- actin interactions and regulates cell width

**Figure 3:**
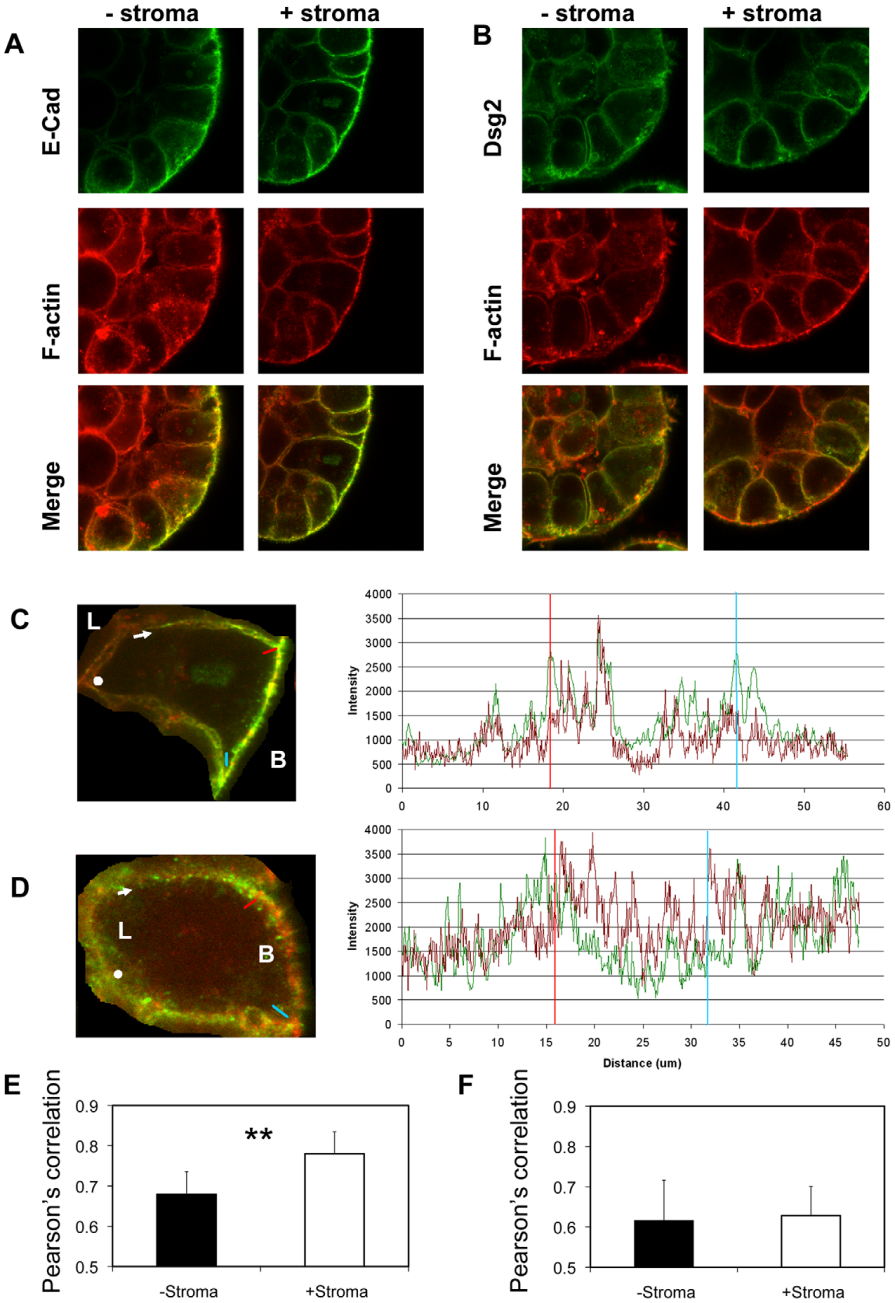
Stroma enhances E-cadherin and F-actin co-localisation. (A and B). 7 day old 3D BPH-1 acini were immunostained for E-cadherin (A, green) or Dsg2 (B, green) and F-actin (red). (C and D). High magnification analysis of co-localisation within single cells indicated that E-cadherin and F-actin (C) co-localised in intense patches along the basolateral membranes, whereas Dsg2 and F-actin did not (D). Using Volocity software, a line was drawn from the arrow to the red mark, blue mark and to the spot in order to measure the green and red fluorescent intensity along the basolateral membrane to produce a graph of fluorescent intensity versus distance from the arrow (with the blue and red markers included for reference). The patterns of red and green fluorescent intensity closely matched for E-cadherin and actin indicating co-localisation, but not for Dsg2 and F-actin. For E-cadherin and F-actin yellow patches of co-localisation clearly correlated with the simultaneous peaks of red and green fluorescence. L =  luminal side of cells, B =  basal edge. (E and F). Comparison of F-actin co localisation with E-cadherin (E) or Dsg2 (F), measured by Pearson's correlation coefficient of acini grown with or without stroma. A paired t-test was used to calculate significance. Each experiment consisted of 10 images. The experiment is typical of three separate stromal cultures, **p<0.005.

In both vertebrates and Drosophila, E-cadherin clusters into discrete regions of cell surface membranes, a process that increases cell surface adhesion [23]. In addition, close interaction of DE-cadherin clusters and F-actin increases junctional stability [24]. To test whether stromal signalling could influence this interaction we examined co-localisation of E-cadherin and F-actin using confocal microscopy of BPH-1 acini. E-cadherin was found to cluster into intense patches and co-localise with F-actin throughout the basolateral membranes of acini, grown with and without stroma (Figures 3A). The Pearson's correlation of co-localisation significantly increased with stromal co-culture (Figure 3C and E). Increased co-localisation was repeated with different primary stromal cultures and different batches of Matrigel (results not shown). Dsg2 and actin were not strongly co-localised in the absence or presence of stroma, this was noted by the diffuse red and green fluorescence confocal images with a lack of yellow co-localisation, accompanied by dissimilar red/green fluorescent intensity vs distance graphs and low Pearson's correlation (Figure 3B, 3D and 3F). Co-localisation was the method of choice for these experiments since calcium switching and pulse chase experiments did not prove practical in 3D models. To confirm the importance of E-cadherin we used an antibody to inhibit function. Acini with stromal co-cultures were grown for 6 days and then antibody added for an additional 24 hours. In the presence of IgG controls 20.3%±12 of the lateral membranes were within 20 nm whilst anti-E-cadherin led to a decrease to 6.9%±6 (p<0.068).

**Figure 4:**
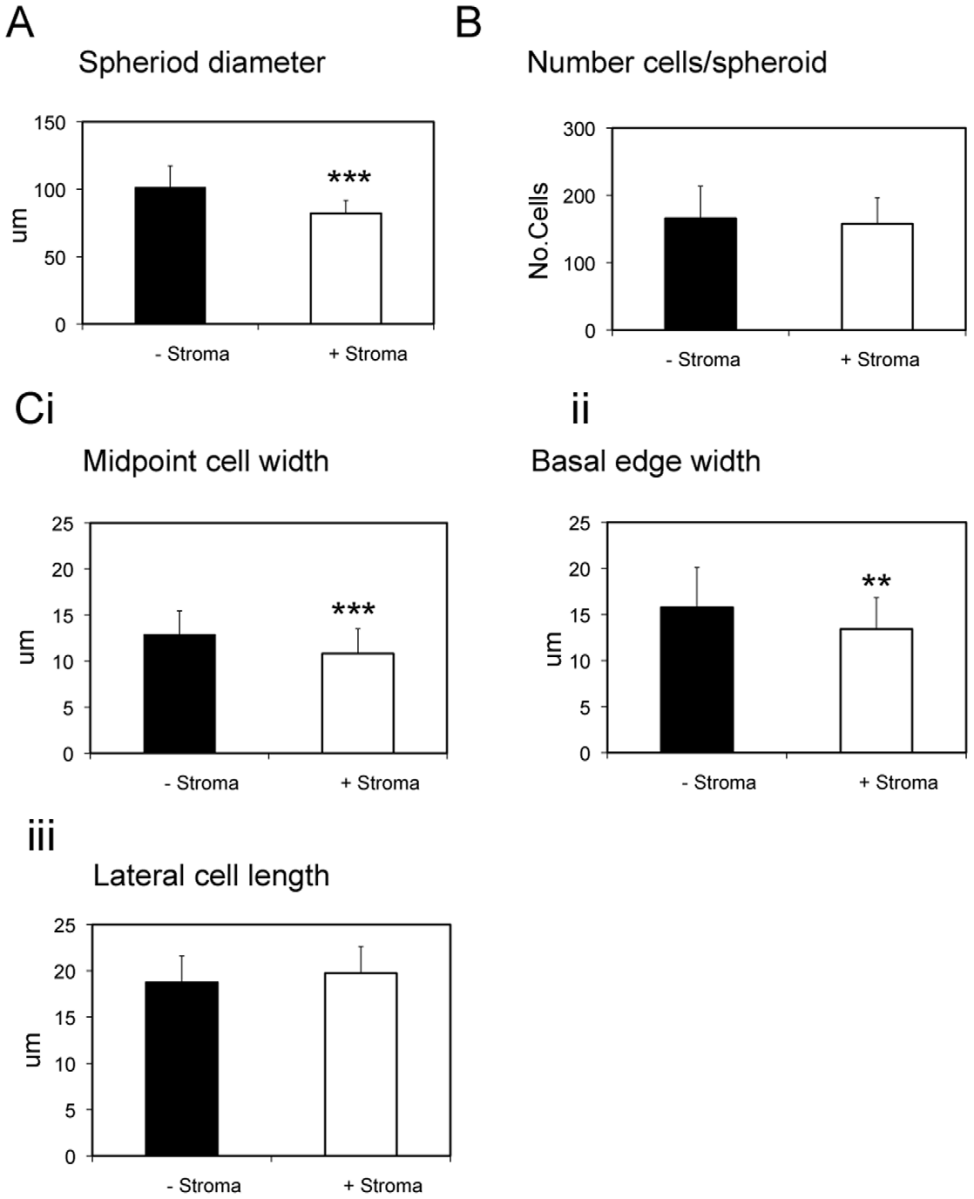
Stroma decreases spheroid size by decreasing cell width. (A) The diameter of 3D BPH-1 acini was measured in the presence or absence of stroma after 7 days in culture. Fifty acini cultured with stroma and 50 cultured without stroma were measured at mid-sections. The data represents five separate experiments, which were performed with different stromal cultures and Matrigel batches. (B) The number of cells within BPH-1 acini cultured with and without stroma were counted, according to Pearson et al. [22]. The cells from 10 spheroids were counted per condition and the experiment was repeated twice. (C) The width of individual cells within acini were measured at the mid-point of the cell (Ci) and at the basal edge of the cell (Cii). The length of individual cells was measured at their longest point (Ciii). Fifty cells were measured from a minimum of 10 spheroids per condition. The data represents three experiments, ***p<0.0005, **p<0.005, *p<0.05 (paired t-test).

Using confocal images, we analysed the influence of stroma on cell shape. Whole BPH-1 acini consistently showed a significant decrease in diameter in the presence of stroma (Figure 4A). We estimated that the smaller acini did not contain fewer cells but the width of the individual cells was significantly decreased (2 µm) and there was no change to their length (Figures 4B and C). A decrease in cell size may be associated with cytoskeletal reorganisation.

### Identification of differentially expressed stromal growth factors and extracellular matrix signals during acinus development

Our previous work has indicated the importance of direct interactions between epithelium and stroma to produce morphological effects (the effects are not achieved using stromal conditioned media), therefore the presence of both cell types is critical [4], [15], [25]. To identify the stromal signals that control actin/cadherin dynamics in our model we performed a microarray study using primary stroma from 7 patients **(Table S1)** cultured with or without 3D BPH-1 acini (Array Express Accession Number: E-MEXP-2657). We identified 4,554 probes with a 1.5 fold (p<0.05) change in expression. Due to the nature of our model system we looked specifically for soluble, paracrine factors which are capable of crossing a culture insert. Therefore, we exported all the soluble growth factor and extracellular matrix molecules (ECM) changing over 1.5 fold (p<0.05) from the data using the gene ontology list for growth factor activity GO:0008083 and ECM GO:0031012. The 20 most differentially expressed genes for each term are represented in **Table 2** **and S2**. Analysis of the ECM genes indicated the importance of collagens, laminins, complement and decorin. However, the role of secreted matrix has not been explored further in this study. Of the secreted growth factors chemokine 12 (CXCL12) showed the greatest change in expression (a 27.9 fold increase) and is a known mediator between prostate epithelial and stromal cells [21]. CXCL12, interacts with components of the TGFβ pathway via the MAPK pathway, and two such genes, BMP5 and TGFβ2, were also up-regulated (8 and 4.4 fold respectively). A splice variant probe for TGFβ2, which codes for a different protein isoform of TGFβ2, was down-regulated. Similarly, TGFβ signalling pathways overlap with fibroblast growth factor (FGF) signalling [26] and FGF10 was up-regulated 3.6 fold whilst FGF13 was down-regulated by 2.56 fold. We analysed the data further using Pathway Express software and identified 18 KEGG pathways unlikely to occur on this probe list by chance **(Table S3)**. Consideration of all these analyses led us to identify three interacting pathways: TGFβ, chemokine (MAPK) and FGF signalling. All three pathways are known to have roles in epithelial morphology. Therefore three genes were selected for further analysis on this basis: TGFβ2, CXCL12 and FGF10.

**Table 2:** Significant differential expression of stromal growth factor genes in response to 3D epithelial cultures.

Probe set	Accession	Gene	Fold	P-value
209687_at	U19495	CXCL12: chemokine 12	27.9	0.0020
208241_at	NM_004495	NRG1: neuregulin 1	10.3	0.0220
210755_at	U46010	HGF: hepatocyte growth factor	9.6	0.0020
205430_at	AL133386	BMP5: bone morphogenetic protein 5	8.0	0.0220
209465_x_at	AL565812	PTN: pleiotrophin	4.6	0.0020
220406_at	NM_003238	TGFB2: transforming growth factor, beta 2	4.4	0.0010
231762_at	NM_004465	FGF10: fibroblast growth factor 10	3.6	0.0320
204220_at	NM_004877	GMFG: glia maturation factor, gamma	3.6	0.0140
219304_s_at	NM_025208	PDGFD: platelet derived growth factor D	3.3	0.0050
206926_s_at	M57765	IL11: interleukin 11	2.89	0.0095
221314_at	NM_005260	GDF9: growth differentiation factor 9	−9.4	0.0454
203821_at	NM_001945	HBEGF: heparin-binding EGF-like growth factor	−6.5	0.0468
207160_at	NM_000882	IL12A: interleukin 12A	−4.6	0.0199
206814_at	NM_002506	NGFB: nerve growth factor, beta polypeptide	−4.1	0.0200
217497_at	AW613387	ECGF1: endothelial cell growth factor 1	−3.8	0.0408
214146_s_at	R64130	PPBP: pro-platelet basic protein	−3.1	0.0379
230686_s_at	AI634662	IL7/SLC13A3: interleukin 7/solute carrier family 13	−2.7	0.0010
209908_s_at	BF061658	TGFB2: Transforming growth factor, beta 2	−2.5	0.0133
205110_s_at	NM_004114	FGF13: fibroblast growth factor 13	−2.6	0.0210
206516_at	NM_000479	AMH: anti-Mullerian hormone	−2.5	0.0411

The probes for growth factor activity genes according to the gene ontology database were exported from the fold change p<0.05 array list. The 20 most differentially expressed genes from the GO:0008083 growth factor activity list were extracted, redundant probes were omitted. Positive values are upregulated and negative values are down regulated.

### Stromal signalling of TGFβ2, CXCL12 and FGF10 to 3D acinus cultures

**Figure 5:**
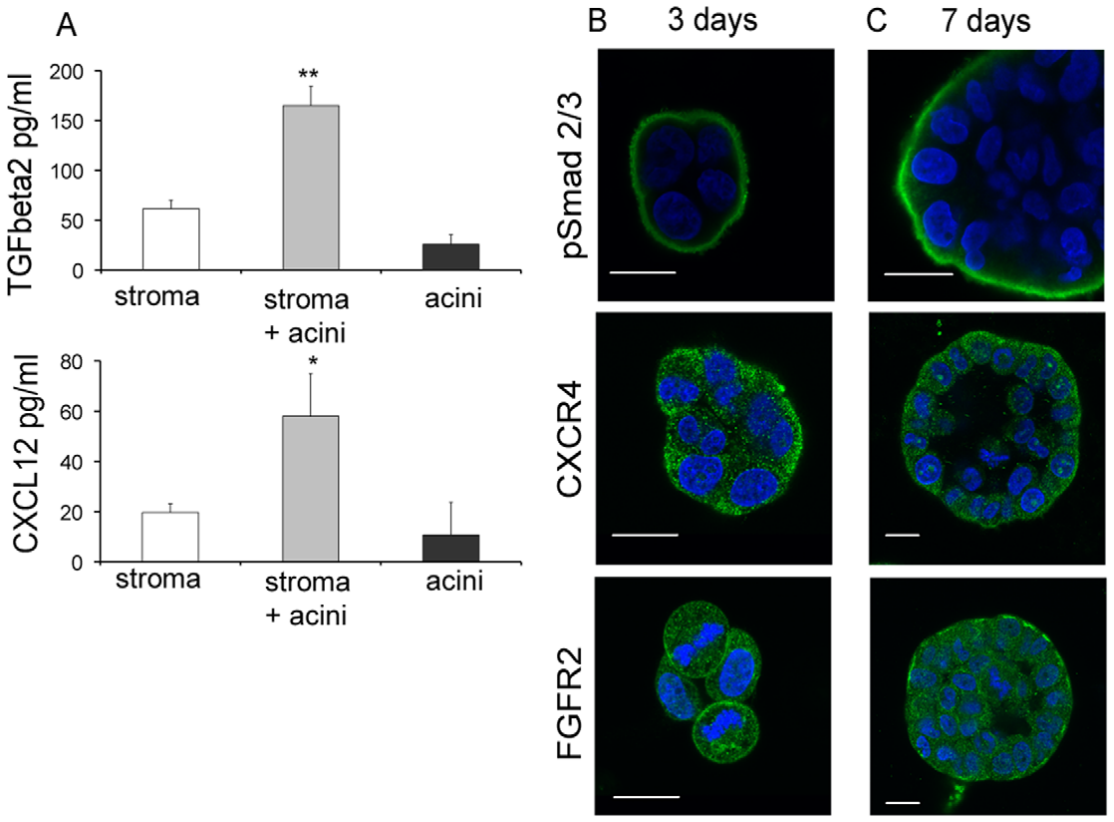
Paracrine signalling of TGFβ2, CXCL12 and FGF10. (A). Protein levels of TGFβ2 or CXCL12 were determined using ELISA. Medium was collected after 3 days growth from stroma or 3D BPH-1 acini cultured alone or in co-culture (n = 6). The datum is typical of results from three separate stroma, ** = p<0.05 (paired t-test). (B and C). 3D BPH-1 acini were fixed at day 3 and day 7 and immunostained for pSmad 2/3, CXCR4 and FGFR2 (green), imaged at mid sections, DAPI (blue), bars  = 20 µm.

To confirm the increased expression of stromal growth factor genes identified in the microarray we determined secreted protein expression of CXCL12, TGFβ2, and FGF10 (Figure 5). Active TGFβ2 secretion was increased approximately 2-fold greater (165 pg/ml) than the sum of TGFβ2 secretions measured in the medium of either cell type cultured alone (stroma, 62 pg/ml; acini, 26 pg/ml). A similar change has been found in monolayer culture [27]. Secreted levels of CXCL12 were low in stroma (20 pg/ml) and acini (11 pg/ml) cultured alone, but comparable to published data [21]. Co-culture increased the combined secreted amount of CXCL12 approximately 2 fold (58 pg/ml). FGF10 secreted levels were too low for detection by ELISA (<100 ng/ml) in stroma cultured alone or in co-culture. However, studies in mice have shown that FGF10 is expressed in prostate mesenchymal stoma, albeit at low levels [16]. Immunofluorescence was used to determine the presence of the CXCL12 receptor, CXCR4, and the FGF10 receptor, FGFR2, on 3D BPH-1 acini, to prove that these growth factors can have a functional consequence on the acini (Figures 5B and 5C). Phosphorylated Smad 2/3 (pSmad2/3) was used as an indicator of TGFβ receptor activity as initially TGFβ2 ligand binds TGFβRII, which then recruits TGFβRI to activate Smad signalling (Figures 5B and 5C). PSmad 2/3 was highly expressed on the basal membrane and in the cytoplasm at days 3 and 7 of culture. This staining was unexpected, pSmad 2/3 is found in the nucleus after TGFβ stimulation in monolayer (Figure S3). Most likely the TGFβ receptor is highly activated on the basal membrane in this model, non-specific staining could account for this, but similar isotype antibodies did not give the same staining pattern in 3D culture, (see CXCR4, FGFR2 and Pearson et al., [22]). CXCR4 and FGFR2 were expressed at days 3 and 7 of culture at the basal membrane and throughout the cytoplasm, indicative of endocytosed, active receptor [28]. In summary, these protein analyses show that TGFβ signalling and CXCL12 signalling can take place between stroma and acini.

### Stromal derived TGFβ increases lateral epithelial cell adhesion in 3D cultures

**Figure 6:**
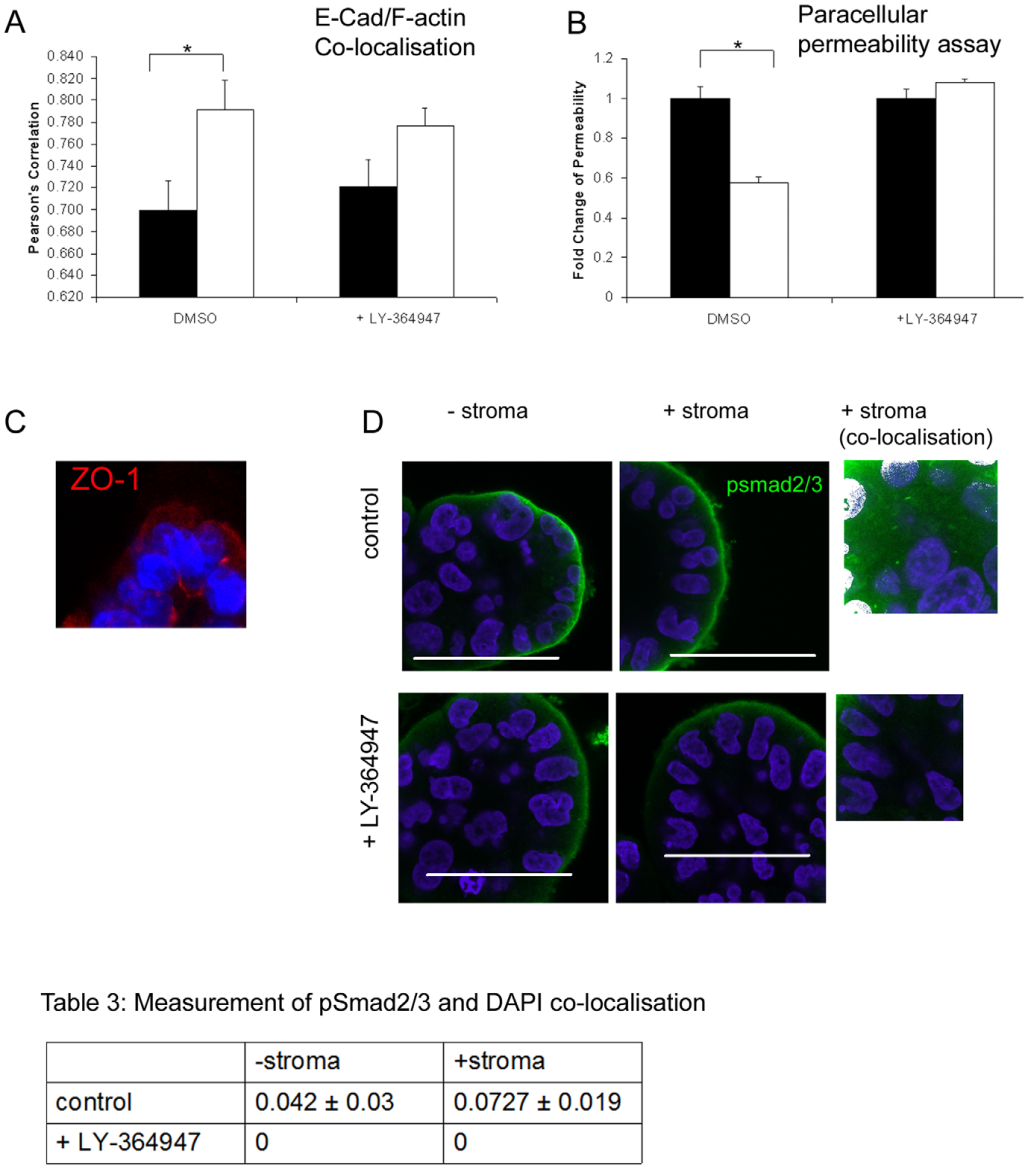
Inhibition of TGFβ signalling disrupts E-cadherin and F-actin co-localisation and cell to cell adhesion. 3D BPH-1 acini were cultured for 7 days in the presence (white bars) or absence of stroma (black bars). Both conditions were treated with 500 nM LY-364947 or DMSO for 7 days. (A). E-cadherin and F-actin co-localisation (n = 10) was measured and the Pearson's correlation plotted *p<0.05. (B). A paracellular permeability assay measured the ability of 70 kDa dextran to diffuse into the lumen of 3D acini. Six wells were measured per assay and the results are representative of two experiments cultured with 2 different stroma. (C). Mid-section of BPH-1 3D acini immunostained for ZO-1 (red), DAPI (blue). Intense staining can be seen at the luminal and lateral membranes. (D) Confocal images of the expression of pSmad 2/3 after 7 days growth. Nuclear staining was not visible without refocusing and recalibrating the confocal microscope away from the basal membrane (+stroma co-localization). Nuclear co-localisation (white) of pSmad 2/3 (green) and DAPI (blue) was only found in the absence of LY-364947. Table 3. Co-localisation was measured (as above) and calculated using Volocity software to obtain the Pearson's correlation (n = 4). All nuclear images were taken at equivalent settings to compare green and blue co-localisation.

To determine the importance of TGFβ2, CXCL12 or FGF10 signalling in E-cadherin and F-actin dynamics we used small molecule inhibitors to inhibit receptor function. Inhibition of TGFβ signalling (500nM LY-364947) blocked significant stimulation of E-cadherin and actin co-localisation, in the presence of stroma (Figure 6A). Inhibition of CXCL12 (1 ug/ml AMD3100) and FGF signalling (500 nM PD173074) had no effect on the levels of co-localisation (results not shown). Inhibitors were used at concentrations known to produce effects in other in vitro studies [29], [30], [31]. To find alternative methods to confirm the effect of TGFβ inhibitors on lateral adhesions we developed a 3D paracellular permeability assay (Figure S4). In the presence of stroma a 70 kDa rhodamine-dextran was significantly excluded from the lumen of spheroids. Addition of LY-364947 inhibited this effect (Figure 6B). A reduction in paracellular fluid movement is associated with a reduction in the space between individual cells and the presence of tight junctions [32]. Although tight junctions were present in BPH-1 spheroids (Figure 6C) and prostate tissue [33] we found no evidence that their expression levels were influenced by stromal co-culture (western blotting and quantitative PCR of ZO-1, ZO-2 and occludin (results not shown). Analysis of the effect of LY-364947 on the proximity of opposing lateral membranes found that on average, 16.2%±3.4 of a lateral cell membrane was within 20 nm of the opposing membrane in control acini (grown with stroma), whilst the addition of LY-364947 decreased these adhesions by one third to 11.2%±6 (p<0.059). The lack of strong inhibition in the presence of inhibitors may indicate that other growth factors are involved. Preliminary experiments using spheroids cultured without stroma but with the addition of TGFβ did not show increased co-localisation of E-cadherin and actin. This may indicate other growth factors are required or the presence and dynamic interaction of stroma is required for this effect. Inhibition of TGFβ signalling was confirmed using pSmad 2/3 expression [34]. pSmad 2/3 could only be imaged at the basal membrane of 3D BPH-1 acini, with or without stroma or TGFβ inhibitor (Figure 6D). As discussed above this staining was either an artefact or we hypothesised that TGFβ receptor was highly activated at the basal membrane and therefore phosphorylated Smad2/3 was highly expressed at the basal membrane. The intense stain masked our ability to image pSmad2/3 in the nucleus. Therefore we carefully imaged the nuclei alone and identified the presence of pSmad 2/3 in 3D cultures with and without stroma (Table 3 and Figure 6D). Nuclear co-localisation of pSmad 2/3 and DAPI was significantly increased in the presence of stroma (p<0.045) and all co-localisation was lost after treatment with LY-364947.

**Table 3:** Measurement of psmad2/3 and DAPI co-localisation.

	−stroma	+stroma
control	0.042±0.03	0.0727±0.019
+LY-364947	0	0

Nuclear co-localisation (white) of pSmad 2/3 (green) and DAPI (blue) was calculated using Volocity software to obtain the Pearson's correlation (n = 4). All nuclear images were taken at equivalent settings to compare green and blue co-localisation.

## Discussion

In this study we aimed to understand how stromal co-culture increased the lateral cell adhesions of prostate epithelial cells grown in 3D Matrigel culture. We discovered that stromal signalling increased the level of E-cadherin and actin co-localisation and did not affect the expression levels of cadherin or desmosomal proteins. These findings reflect earlier work which found that adhesion is controlled by the stability of cadherin/actin interactions [35], [36]. Our results show for the first time that stromal cells can control E-cadherin/actin dynamics in epithelial cells. Using the model proposed by Cavey et al. [24] our results may suggest that stromal-derived TGFβ can increase either the stability of interaction or the frequency of interaction of E-cadherin and actin. Cavey et al. [24] demonstrated that actin patches increase the stability of spot adherens junctions and, consistent with this, we found that E-cadherin and actin co-localised in discrete patches throughout the basolateral membrane of prostate acini. Therefore, it is likely that stroma provides signals to stabilise adherens junctions to increase lateral cell adhesions. The precise nature of the epithelial mechanisms involved in the increased lateral cell adhesion has not yet been fully understood, but may prove to be complex and will undoubtedly involve several pathways. Indeed our preliminary work has indicated that stroma could also decrease paracellular permeability and that inhibition of TGFβ reverses this effect. Paracellular permeability to fluorescent dextrans is commonly used to assess the function of tight junctions [32]. Therefore stroma (and TGFβ) may influence both cadherin and tight junction adhesions. In other epithelial cell types TGFβ signalling can increase tight junction adhesion through the proteins FLRT3, Rnd1 and claudin and increase the association of cadherin with β-catenin to promote junction formation [37], [38], [39], [40]. However, until this study, TGFβ signalling had not been found to have a role in cadherin/actin interactions. Further analyses will be required to elucidate the mechanism of TGFβ action (e.g. development of SiRNA for 3D culture or dominant negatives are required) and to determine whether other factors are important.

Microarray analysis also highlighted the differential expression of many stromal extracellular matrix proteins that may act as paracrine signals between the stroma and the epithelium, but which have not been pursued here. Interaction between matrix and lateral cell adhesions was recently demonstrated by Dzamba et al. [41], who showed that non-canonical Wnt signalling engaged with cadherins leading to the reorganisation of actin, increased intracellular tension and transmission of the signal to the extracellular matrix via integrins. The presence of actin/cadherin complexes at the basal edge of lateral membranes in our model could indicate a similar actin-driven mechanism here, whereby increased actin polymerisation could explain the reduction in cellular width. The observation that E-cadherin was highly expressed on cellular protrusions from the basal membrane may also indicate matrix driven mechanisms or represent a culture artefact. Such protrusions are consistent with the expression of E-cadherin on filopodia-like extensions in keratinocytes, which are thought to seek adhesions with neighbouring cells [42].

Intriguingly, the ability of stroma to signal an increase in epithelial adhesion, in 3D, is in direct conflict with the previously observed ability of stromal cultures to induce epithelial-mesenchymal transition or scattering of epithelial cells in monolayer [43], an effect we have previously observed [5]. Scattering in response to stroma provides a mechanism for tumour epithelial cells to become more invasive. However, in normal adult tissue, stroma clearly does not act to decrease epithelial cell adhesion and reduce tissue integrity. Our model may therefore replicate the normal tissue environment more accurately than monolayer cell culture models. If epithelial cells are exposed to the same combinations of culture medium and stromal growth factors in 3D and monolayer [5] culture, the difference in response to these factors must therefore lie in the physical nature of the culture models. Tissue culture plastic or glass, the normal substrata for the growth of cells in monolayer culture, are rigid. In 3D culture the matrix surrounding the cells is deformable, as in vivo, permitting cells and cell populations to adopt a more tissue-like morphology. The difference in response of epithelial cells to stroma when cultured in monolayer compared to 3D reflects both the influence of the signals (matrix and growth factor signals) and the physical characteristics of the microenvironment, such as deformability and tensile strength. The conflict of our findings and those of EMT are highlighted by the upregulation of genes associated with EMT in our microarray analysis, such as hepatoctye growth factor (HGF) (+9.6 fold). During EMT, HGF functions to dissociate epithelial cell adhesions (scattering) and increase migration in monolayer culture [44], but clearly HGF did not induce scattering in 3D. Interestingly, in 3D collagen culture HGF induces branching not scattering [45] using a mechanism of partial EMT [46]. EMT in monolayer is associated with the increased production of cellular protrusions or adhesion puncta at the cell membrane, and these were clearly seen in 3D. Cellular protrusions are also required to aid the ability of two cells with contacting membranes to interdigitate (the zippering model) and form new mature membrane contacts [28]. Therefore, it can be hypothesised that stroma increases cellular protrusions in both models, but in 3D additional factors dictate that increased protrusions promote adhesion. This may provide a mechanism for stroma to maintain tissue integrity in adult epithelial tissue. Breast epithelial cell cultures grown in 3D (Matrigel and collagen) also produce acini or ductal structures in the presence of stromal cells, though the effect of co-culture on intercellular adhesions was not closely investigated [47], [48].

Several stromal growth factors were found to be significantly differentially expressed when stroma was co-cultured with epithelia in 3D. It is likely that growth factors other than TGFβ and/or matrix molecules will be important in the control of epithelial morphology, and should be explored in the future. A potential role for FGFs should not be discounted since they have important functions in embryonic prostate and embryonic epithelial morphology via cytoskeletal remodelling as well as in preserving membrane integrity [49], [50]. Other significantly regulated pathways include the Wnt/wingless pathway and notch, which have important functions in regulation of cadherin adhesions [51], [52].

In summary, we found that stroma increased lateral cell adhesions between adult human epithelial cells in 3D culture. Increased lateral adhesion is likely to be produced by changes to actin/cadherin dynamics signalled by TGFβ. This signalling mechanism increased membrane co-localisation of actin and cadherin, decreased cellular width and decreased paracellular permeability. We propose that such a mechanism could be important in maintaining tissue integrity in normal adult tissues. 3D modelling provides a useful system to investigate the control of adult epithelial morphology, and should provide a useful tool to confirm developmental mechanisms identified in lower organisms.

## Materials and Methods

### 3D Matrigel culture and inhibition studies

Primary stromal cells were derived from BPH prostate tissue. The use of human tissue and patient consent procedures were approved by York Research Ethics Committee, (YREC Reference 91/7/6) and Hull and East Riding Local Research Ethics Committee (REC Reference Number 07/H1304/121). Tissues were obtained from York District Hospital, York and Castle Hill Hospital, Hull, UK. All patients who provided tissue gave their written consent. Tissues were given a unique identification number which was stored with the consent forms at participating hospitals, whilst documentation of tissue processing, experimentation and storage occurred at the YCR Cancer Research Laboratory. BPH-1 cells and primary cultures were cultured in 3D as previously described [4], [22]. Briefly, primary stromal cells were pre-seeded onto culture inserts (Millipore) and grown in RPMI 1640 plus 10% FCS and 2 mM L-glutamine for 3 days prior to co-culture. BPH-1 cells were seeded onto 24-well plates (Nunc) or 8-well chamber slides (Nunc) in a solution of 4% Matrigel in the presence of KEF2 (keratinocyte-serum free medium supplemented with 5 ng/ml recombinant epidermal growth factor, 1 ng/ml basic fibroblast growth factor, 2% FCS, 2 mM L-glutamine, 10 nM dihydrotestosterone and 10 nM β-estradiol). After plating epithelia into Matrigel stromal inserts were added to the cultures, media was replenished every 2–3 days. Primary stromal cultures were used at passage 1–3. Spheroids for RNA extraction, RT-PCR and western blotting were isolated from the Matrigel using BD Cell recovery solution (Becton Dickinson, Plymouth, UK). For inhibition studies, LY-364947, AMD3100, PD173074 or vehicle (Sigma) were added from day 0, media was replenished every 2–3 days.

### TEM and Imaging

TEM, tissue sections and 3D cultures were stained and imaged as described [4], [22], [53]. Antibody dilutions are given in Tables S4 and S5. ELISA kits for CXCL12 and active TGFβ2 were purchased from R&D systems (Abingdon, UK) and for FGF10, from Antigenix America (New York, USA). All were used according to manufacturer's protocols.

For co-localisation studies twelve-bit images were collected, using the same palette between images, at optimal settings, with 63 x oil immersion lens (NA 1.4). Images were collected at mid-section through the acini and analysed using Volocity Version 5.0 [54]. Images were cropped to include 3–5 whole cells. A region of interest (ROI) was drawn around the in-cell background of one cell from each image, and used to subtract background pixels for that respective image. The resultant Pearson's correlation is a measure of the number of co-localised red and green pixels, calculated using the Volocity software.

### Western Blotting

Proteins were extracted and separated on 10% SDS-polyacrylamide gels according to Pearson et al., [22]. Membranes were blocked with 5% BSA and then incubated with primary and secondary antibodies as indicated in **Tables S4 and S5**. Fluorescence intensity was measured using Image J [55] and normalised to β-actin expression.

### Reverse transcriptase-PCR (RT-PCR) and Quantitative real-time PCR (TaqMan)

RNA was prepared using Illustra RNA Spin mini kit (GE Healthcare). Reverse transcription was performed with random hexamers (SuperScript™II, Invitrogen).

For RT-PCR: cDNA was amplified with primer pairs (Table S6) in a GeneAmp PCR System 9700 thermal cycler (Applied Biosystems) according to the protocol: 5 minutes at 95°C; 35 cycles of 30 s 95°C, 30 s 50–55°C and 30 s per 500 bp at 72°C; 5 minutes at 72°C. Amplified cDNA fragments were separated on a 1% agarose gel and revealed after staining with ethidium bromide.

Quantitative real time PCR oligonucleotide primers and probes (Table S7) were designed using Primer Express 3.0 (Applied Biosystems). Reactions used Taqman one-step mastermix kit (Applied biosystems) and 100 ng total cDNA. Target mRNA levels were detected using the ABI prism 7700 sequence detection system (Applied Biosciences) and normalized to GAPDH using the ddCt relative quantification method [56]. The real time PCR conditions were as follows: 1 cycle at 50°C for 2 min, 1 cycle at 95°C for 10 min, 40 cycles at 95°C for 15 s, and 60°C for 1 min. Assays consisted of three technical replicates and three biological replicates.

### Microarray analysis

RNA was extracted from stromal cells cultured either in the presence or absence of acini (n = 7). Samples were analysed using Affymetrix Human Genome U133 Plus 2.0 chips (Affymetrix Inc., Santa Clara, CA). The cRNA synthesis of the samples was carried out according to the manufacturer's protocol. The fluorescence intensity for each chip was captured with an Affymetrix GeneChip Scanner 3000. Affymetrix Microarray Suite version 5.0 was used to quantify each chip. The raw data (CEL) files, were loaded into the DNA-chip analyser software (dChip) version Feb 2009 [57]. Normalisation was carried out using Invariant Set Normalization method and probe expression values were then calculated using the perfect match (PM)-only model. Unsupervised hierarchical clustering was performed as described [58]. Three comparison criteria were applied to detect differentially expressed genes by model based expression: 1) the fold change between the group means was chosen to exceed 1.5 fold; 2) absolute difference between the two groups means >50 to eliminate background; 3) a p-value of 0.05 for Welch's modified 2-sample paired t-test, adjusted to compensate for multiple testing using False discovery rate (1000 permutations). The raw data is deposited in ArrayExpress (E-MEXP-2657). All data is MIAME compliant. Functional analysis was performed on the 1.5, p<0.05 probe list using Pathway-Express [59].

### Paracellular permeability assay

We adapted the assay of Matter et al., [32]. 1 mg/ml 4 kDa FITC or 70 kDa rhodamine dextran (Sigma) was added to BPH-1 spheroids in growth medium for 24 hours, 37°C, 5% CO_2._ Spheroids were harvested using BD cell recovery solution, on ice, and broken open with 1% (v/v) trypsin to release the luminal dyes. Aliquots were prepared in 96 well plates and the level of dye fluorescence was measured in a BMG Labtech POLARstar OPTIMA fluorescent plate reader. Using this method we have shown that in the presence of stroma the level of 70 kDa rhodamine reaching the lumen drops to 9% and 4 kDa FITC drops to 29% **(Figure S4)**, indicating that this a good method to evaluate paracellular permeability in 3D cultures.

## Supporting Information

Figure S1 **RNA expression of desmosomal isoforms in BPH-1 spheroids.** A) Semi-quantitative RT-PCR showing the relative expression of RNA isolated from BPH-1 spheroids for 7 days culture. The fold difference in gene expression was expressed relative to GAPDH for equal loadings of 250ng cDNA. Error bars represent standard deviation of three independent experiments. B) RT-PCR analysis of monolayer (mono) and 3D cultures of BPH-1 cells grown with (3D+S) or without stroma to show the presence or absence of Dsg 1, Dsg 2, Dsg 3 or E-cadherin. Hacat cells grown in monolayer were included as a control for Dsg 1 (Hacat).
(TIF)

Figure S2 **Imaging E-cadherin and desmosomes in BPH-1 acini.** A) E-cadherin expression (red) at the basal edge of a BPH-1 acinus, showing intense staining of cellular protrusions. Confocal microscopy images, x63 magnification, bars  = 20 µm. B) desmoglein expression (red) in BPH-1 acini was punctate manner. Confocal microscopy images, ×63 magnification, bars  = 20 µm, L  =  lumen. C) Time course analysis of Dsg 2 expression (red) in BPH-1 acini, nuclei were counterstained with DAPI. Representative images are shown that cross section through the middle of developing acini. Arrows indicate the expression of functional Dsg 2 at day 4. Confocal microscopy images, x20 magnification, bars  = 50 µm.
(TIF)

Figure S3 **Phosphorylated Smad2/3 translocates to the nucleus in response to TGFβ2 ligand.** BPH-1 cells grown in monolayer were treated with 5 ng/ml TGFβ2 (Sigma) ligand for 24 hours, a dose known to be effective for cell culture [60]. Before treatment phosphorylated smad 2/3 (green) was found in the cytoplasm and after treatment was found in the nucleus (DAPI-blue).
(TIF)

Figure S4 **Dye exclusion from the lumen of BPH-1 spheroids.** BPH-1 cells were grown in Matrigel with or without stroma for 7 days. FITC 4 kDa dye (black bars) or rhodamine 70 kDa dye (clear bars) was added for 24 hours, after which spheres were removed from matrigel and trypsinised to release the dyes within the lumen. Measurement of dyes was performed with a plate reader and expressed as a percentage of that measured in spheres grown without stroma (n = 3). In the presence of stroma 91% of rhodamine dye was excluded from the lumen and 71% of FITC dye. This indicates that stroma increases the junctional contacts between cells and is able to exclude dyes in a size selective manner. The pattern of exclusion was repeated in two separate experiments with different stroma.
(TIF)

Table S1 **Tissue sample details.** The sample identity (ID) is listed, along with the diagnosis of the patient (BPH, benign prostatic hyperplasia), age and batch of Matrigel.
(DOC)

Table S2 **Significant differential expression of stromal extracellular matrix genes in response to 3D epithelial cultures.** The probes for extracellular matrix genes according to the gene ontology database were exported from the fold change p<0.05 array list. The 19 most differentially expressed genes from the GO:0031012 extracellular matrix genes list were extracted (there were only 9 extracellular matrix probes down-regulated on the entire list), redundant probes were omitted. Positive values are upregulated and negative values are down regulated.
(DOC)

Table S3 **Functional pathway analysis using Pathway Express to determine significant KEGG pathways occurring in the stroma in response to the presence of 3D BPH-1 cells cultured in Matrigel.** The 1.5 fold change p<0.05 list of probe changes was used to determine the most significant KEGG pathways changing in stroma in the presence of BPH-1 spheroids. Pathways are ranked according to impact factor and the number of genes changing in each pathway shown. These are divided into up-regulated and down-regulated genes and the fold change shown.
(DOC)

Table S4 **Primary antibodies and dilutions for immunofluorescence and Western Blotting.**
(DOC)

Table S5 **Secondary antibodies and dilutions for immunofluorescence and Western blotting.**
(DOC)

Table S6 **Primers for RT-PCR.**
(DOC)

Table S7 **Primers and probes for real time RT-PCR.**
(DOC)
