# Supplementary figures and images for: Stroma Regulates Increased Epithelial Lateral Cell Adhesion in 3D Culture: A Role for Actin/Cadherin Dynamics
Source: PLoS One. 2011 Apr 18;6(4):e18796. doi: 10.1371/journal.pone.0018796 (PMC3078910; doi:10.1371/journal.pone.0018796)

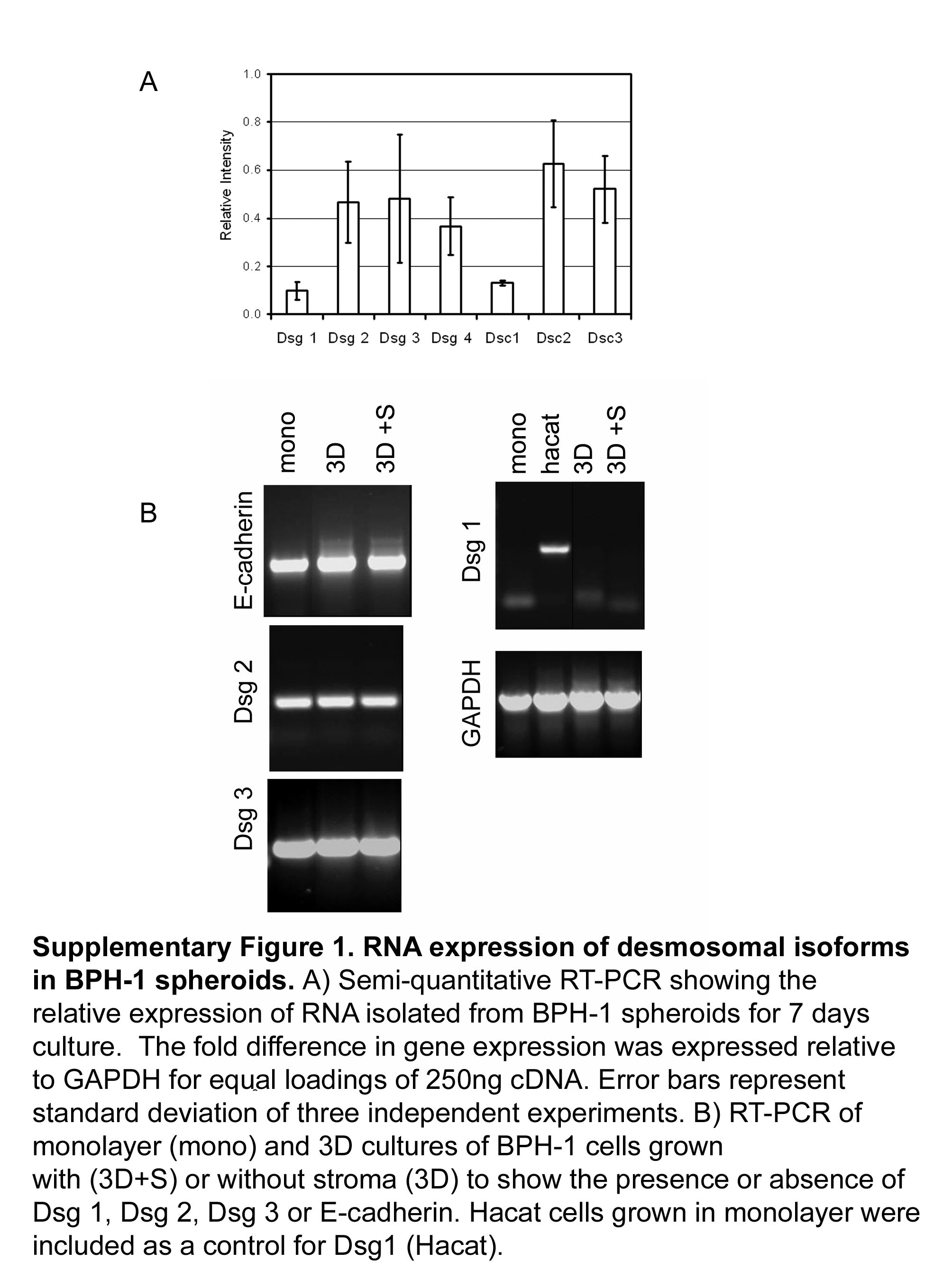

Supplement: Figure S1 — RNA expression of desmosomal isoforms in BPH-1 spheroids. A) Semi-quantitative RT-PCR showing the relative expression of RNA isolated from BPH-1 spheroids for 7 days culture. The fold difference in gene expression was expressed relative to GAPDH for equal loadings of 250ng cDNA. Error bars represent standard deviation of three independent experiments. B) RT-PCR analysis of monolayer (mono) and 3D cultures of BPH-1 cells grown with (3D+S) or without stroma to show the presence or absence of Dsg 1, Dsg 2, Dsg 3 or E-cadherin. Hacat cells grown in monolayer were included as a control for Dsg 1 (Hacat). (TIF) [file pone.0018796.s001.tif]

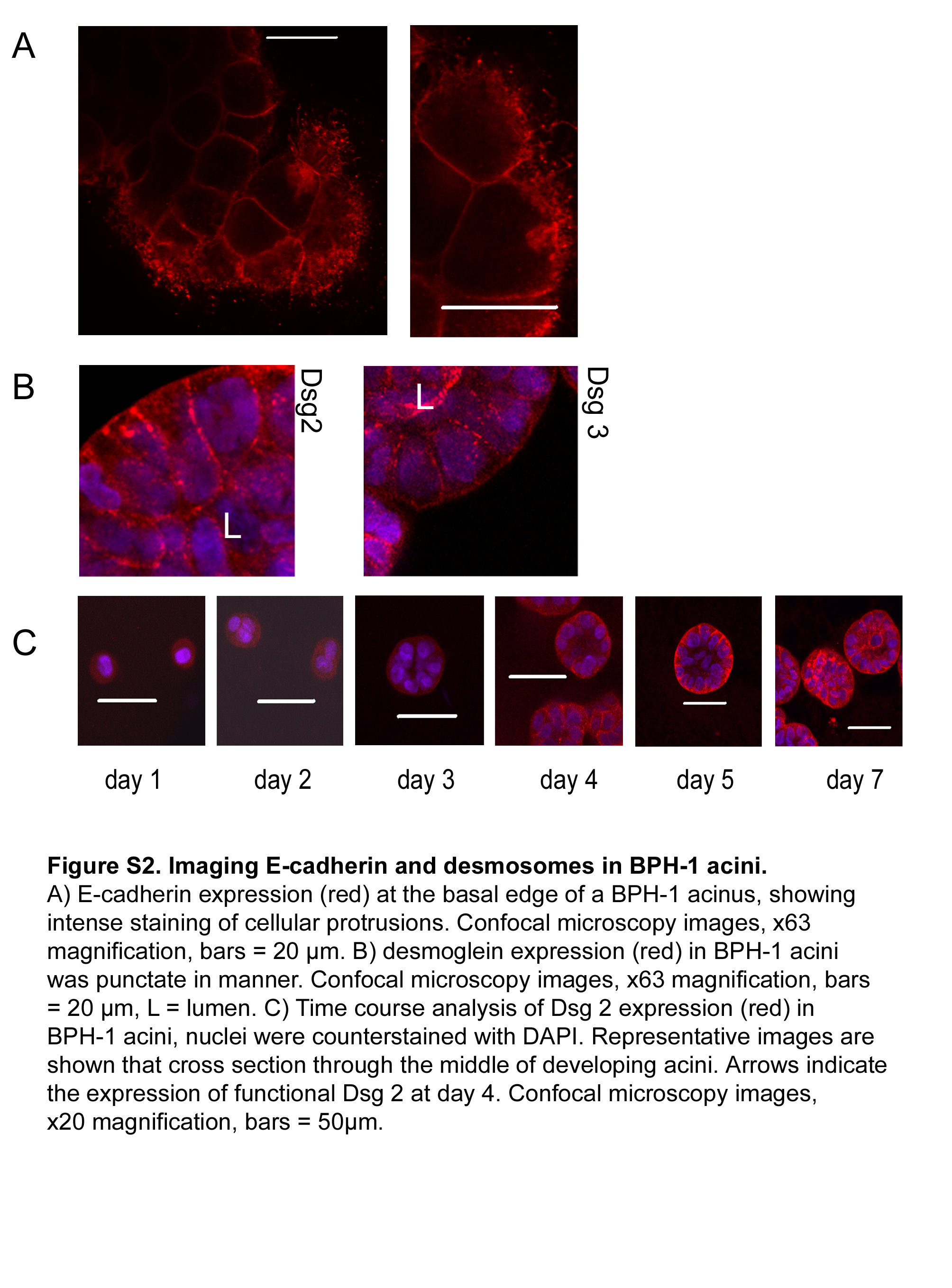

Supplement: Figure S2 — Imaging E-cadherin and desmosomes in BPH-1 acini. A) E-cadherin expression (red) at the basal edge of a BPH-1 acinus, showing intense staining of cellular protrusions. Confocal microscopy images, x63 magnification, bars = 20 µm. B) desmoglein expression (red) in BPH-1 acini was punctate manner. Confocal microscopy images, ×63 magnification, bars = 20 µm, L = lumen. C) Time course analysis of Dsg 2 expression (red) in BPH-1 acini, nuclei were counterstained with DAPI. Representative images are shown that cross section through the middle of developing acini. Arrows indicate the expression of functional Dsg 2 at day 4. Confocal microscopy images, x20 magnification, bars = 50 µm. (TIF) [file pone.0018796.s002.tif]

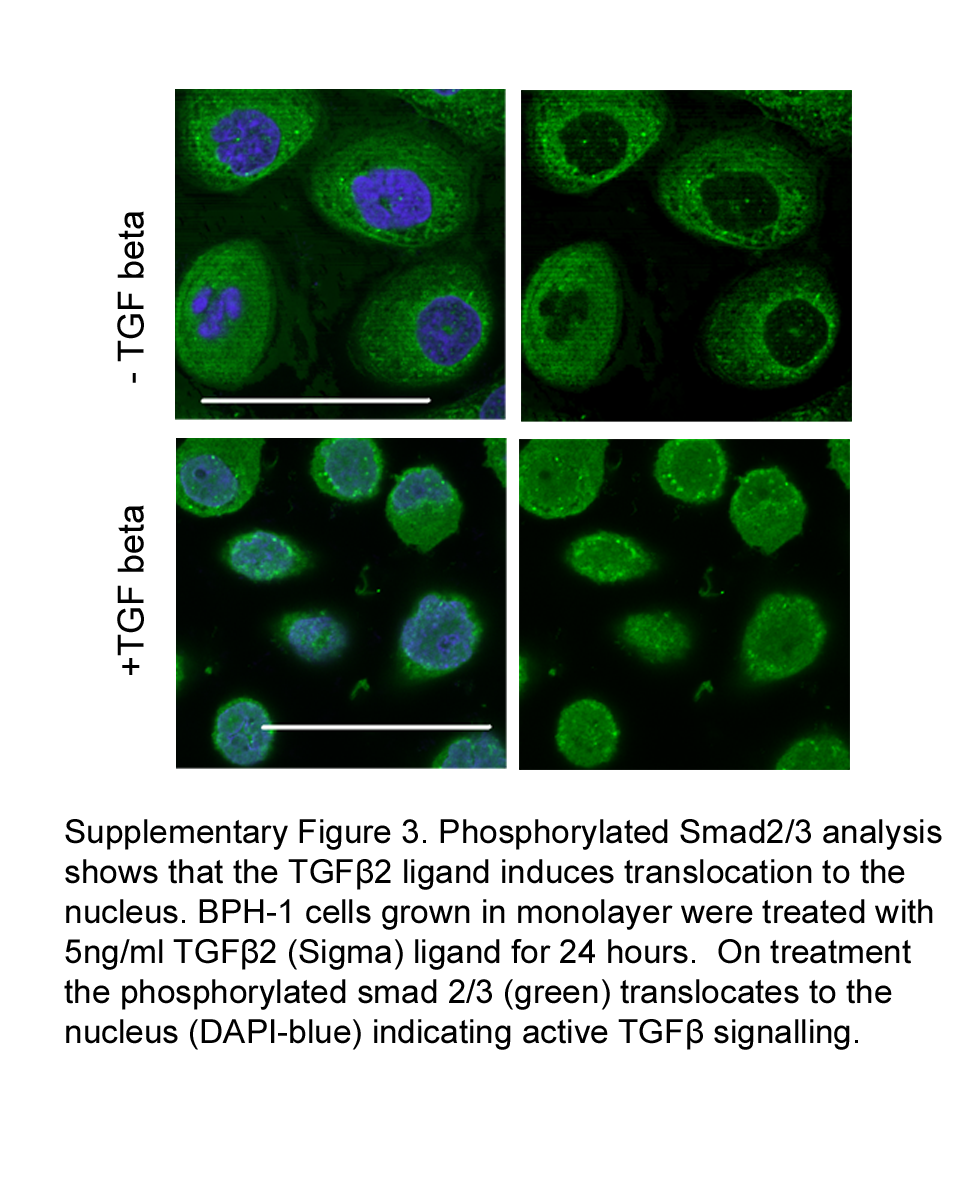

Supplement: Figure S3 — Phosphorylated Smad2/3 translocates to the nucleus in response to TGFβ2 ligand. BPH-1 cells grown in monolayer were treated with 5 ng/ml TGFβ2 (Sigma) ligand for 24 hours, a dose known to be effective for cell culture [60]. Before treatment phosphorylated smad 2/3 (green) was found in the cytoplasm and after treatment was found in the nucleus (DAPI-blue). (TIF) [file pone.0018796.s003.tif]

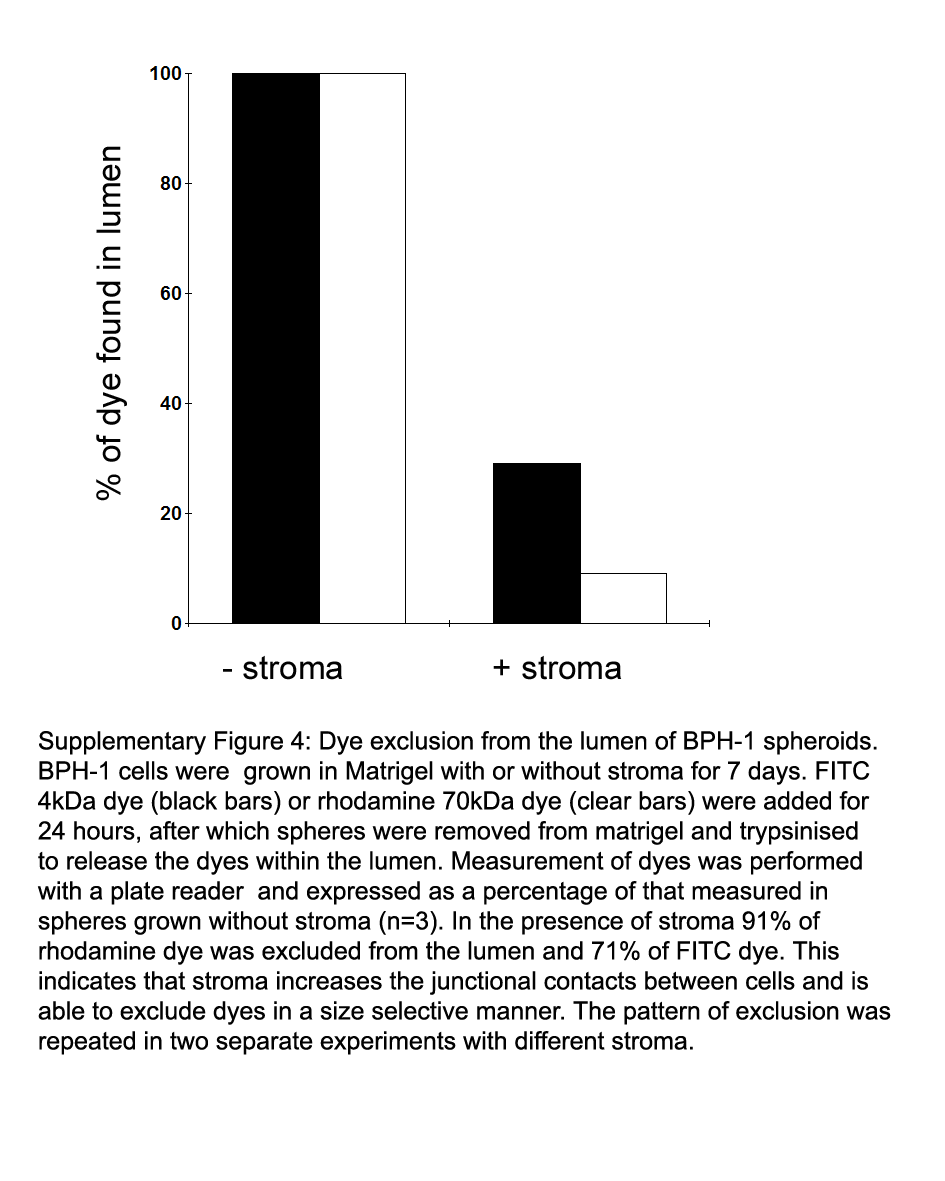

Supplement: Figure S4 — Dye exclusion from the lumen of BPH-1 spheroids. BPH-1 cells were grown in Matrigel with or without stroma for 7 days. FITC 4 kDa dye (black bars) or rhodamine 70 kDa dye (clear bars) was added for 24 hours, after which spheres were removed from matrigel and trypsinised to release the dyes within the lumen. Measurement of dyes was performed with a plate reader and expressed as a percentage of that measured in spheres grown without stroma (n = 3). In the presence of stroma 91% of rhodamine dye was excluded from the lumen and 71% of FITC dye. This indicates that stroma increases the junctional contacts between cells and is able to exclude dyes in a size selective manner. The pattern of exclusion was repeated in two separate experiments with different stroma. (TIF) [file pone.0018796.s004.tif]
