# Supplementary material for: Stroma Regulates Increased Epithelial Lateral Cell Adhesion in 3D Culture: A Role for Actin/Cadherin Dynamics
Source: PLoS One. 2011 Apr 18;6(4):e18796. doi: 10.1371/journal.pone.0018796 (PMC3078910; doi:10.1371/journal.pone.0018796)
Supplement: Table S1 — Tissue sample details. The sample identity (ID) is listed, along with the diagnosis of the patient (BPH, benign prostatic hyperplasia), age and batch of Matrigel. (DOC) [file pone.0018796.s005.doc]

**Supplementary Table S1: Tissue sample details.**

| Sample ID | Diagnosis | Age patient | Matrigel batch |
| --- | --- | --- | --- |
| Exp 34 | BPH | 59 | 4760 |
| Exp 42 | BPH | 75 | 4760 |
| Exp 49 | BPH | 75 | A1005 |
| Exp 53 | BPH | 77 | A1005 |
| Exp 54 | BPH | 67 | A1005 |
| Exp 55 | BPH | 83 | A1005 |
| Exp 56 | BPH | 72 | A1005 |

The sample identity (ID) is listed, along with the diagnosis of the patient (BPH, benign prostatic hyperplasia), age and batch of Matrigel.
