# Supplementary material for: Stroma Regulates Increased Epithelial Lateral Cell Adhesion in 3D Culture: A Role for Actin/Cadherin Dynamics
Source: PLoS One. 2011 Apr 18;6(4):e18796. doi: 10.1371/journal.pone.0018796 (PMC3078910; doi:10.1371/journal.pone.0018796)
Supplement: Table S2 — Significant differential expression of stromal extracellular matrix genes in response to 3D epithelial cultures. The probes for extracellular matrix genes according to the gene ontology database were exported from the fold change p<0.05 array list. The 19 most differentially expressed genes from the GO:0031012 extracellular matrix genes list were extracted (there were only 9 extracellular matrix probes down-regulated on the entire list), redundant probes were omitted. Positive values are upregulated and negative values are down regulated. (DOC) [file pone.0018796.s006.doc]

**Supplementary Table S2: Significant differential expression of stromal extracellular matrix genes in response to 3D epithelial cultures**

| Probe set | Accession | Gene | Fold | P-value |
| --- | --- | --- | --- | --- |
| 232458_at | AU146808 | COL3A1: Collagen, type III, alpha 1 | 19.9 | 0.0066 |
| 219025_at | NM_020404 | CD248: endosialin | 13.7 | 0.0029 |
| 204163_at | NM_007046 | EMILIN1: elastin microfibril interfacer 1 | 12.1 | 0.0006 |
| 205158_at | NM_002937 | RNASE4: ribonuclease, RNase A family, 4 | 11.5 | 0.0036 |
| 202311_s_at | AI743621 | COL1A1: collagen, type I, alpha 1 | 10.7 | 0.0037 |
| 209395_at | M80927 | CHI3L1: chitinase 3-like 1 | 10.2 | 0.0320 |
| 212489_at | AI983428 | COL5A1: collagen, type V, alpha 1 | 8.5 | 0.0026 |
| 213992_at | AI889941 | COL4A6: collagen, type IV, alpha 6 | 8.2 | 0.0017 |
| 204136_at | NM_000094 | COL7A1: collagen, type VII, alpha 1 | 6.2 | 0.0002 |
| 242605_at | AI453137 | DCN: Decorin | 5.5 | 0.0054 |
| 206727_at | K02766 | C9: complement component 9 | -14.3 | 0.0377 |
| 1557558_s_at | BE675718 | MATN1: Matrilin 1, cartilage matrix protein | -7.4 | 0.0100 |
| 210910_s_at | BC000487 | POMZP3: POM (POM121 homolog, rat) and ZP3 fusion | -3.5 | 0.0011 |
| 231766_s_at | U73778 | COL12A1: collagen, type XII, alpha 1 | -3.2 | 0.0178 |
| 208086_s_at | M92650 | DMD: dystrophin | -3.1 | 0.0055 |
| 223315_at | AF278532 | NTN4: netrin 4 | -3.0 | 0.0000 |
| 227048_at | AI990816 | LAMA1: laminin, alpha 1 | -2.8 | 0.0315 |
| 231511_at | AI681772 | FRAS1: Fraser syndrome 1 | -2.3 | 0.0046 |
| 229103_at | AA463626 | WNT3: wingless-type MMTV integration site family, member 3 | -2.0 | 0.0007 |

The probes for extracellular matrix genes according to the gene ontology database were exported from the fold change p<0.05 array list. The 19 most differentially expressed genes from the GO:0031012 extracellular matrix genes list were extracted (there were only 9 extracellular matrix probes down-regulated on the entire list), redundant probes were omitted. Positive values are upregulated and negative values are down regulated.
