# Supplementary material for: Stroma Regulates Increased Epithelial Lateral Cell Adhesion in 3D Culture: A Role for Actin/Cadherin Dynamics
Source: PLoS One. 2011 Apr 18;6(4):e18796. doi: 10.1371/journal.pone.0018796 (PMC3078910; doi:10.1371/journal.pone.0018796)
Supplement: Table S3 — Functional pathway analysis using Pathway Express to determine significant KEGG pathways occurring in the stroma in response to the presence of 3D BPH-1 cells cultured in Matrigel. The 1.5 fold change p<0.05 list of probe changes was used to determine the most significant KEGG pathways changing in stroma in the presence of BPH-1 spheroids. Pathways are ranked according to impact factor and the number of genes changing in each pathway shown. These are divided into up-regulated and down-regulated genes and the fold change shown. (DOC) [file pone.0018796.s007.doc]

**Supplementary Table S3: Functional pathway analysis using Pathway Express to determine significant KEGG pathways occurring in the stroma in response to the presence of 3D BPH-1 cells cultured in Matrigel.**

| Pathway Name  (KEGG ID) | Impact Factor | Input genes/ genes in pathway | Genes Up-regulated | Genes Down-regulated |
| --- | --- | --- | --- | --- |
| Leukocyte transendothelial migration (04670) | 149.16 | 13/114 | CXCL12,VCAM1,CTNNB1,  RAP1A,RAC2, F11R,PRKCG,PECAM1, | CLDN1,VCL,GNAI1,EZR,  CLDN15, |
| Cell adhesion molecules (CAMs) (04514) | 141.51 | 11/127 | VCAM1,ITGB8,CNTNAP1,  SDC2,F11R,PECAM1,SDC3 | CLDN1,PVR,CLDN15,PTPRF |
| Adherens junction (04520) | 20.12 | 16/76 | CTNNB1,SMAD3,SNAI2,RAC2, ERBB2 | ACVR1C,VCL,SRC,MET,  PARD3,PTPRF,EP300,CREBBP,  PTPRJ, ACP1,CSNK2A1 |
| Circadian rhythm (04710) | 12.53 | 02/12 | CSNK1E | CRY1 |
| ECM-receptor interaction (04512) | 10.63 | 26/83 | COL1A1,ITGB8,COL3A1,  COL1A2,LAMA2,ITGB5,CD36,  ITGA2,FN1,THBS1,COL5A1,  COL4A2,SDC2,LAMA5,  COL5A2,COL4A6,SV2A, COL6A2,COL4A1,LAMB1,  CD47, SDC3,THBS3,ITGA5 | ITGA5,THBS3,SDC3,CD47,  LAMB,COL4A1,COL6A2,SV2A,COL4A6,COL5A2,LAMA5,SDC2,COL4A2,COL5A1,THBS1,FN1,  ITGA2,CD36,ITGB5,LAMA2,  COL1A2,COL3A1,ITGB8,  COL1A1 |
| Basal cell carcinoma(05217) | 10.55 | 13/54 | WNT16,WNT4,WNT2,CTNNB1, FZD1,WNT5A,WNT2B,STK36, SUFU | GSK3B,FZD6,APC2,WNT3 |
| Focal adhesion(04510) | 9.84 | 48/199 | COL1A1,DOCK1,CTNNB1,  PDGFRB,HGF,ITGB8,MAPK10,PDGFRA,COL3A1,COL1A2,  LAMA2,ITGB5,RAP1A,ITGA2,PDGFD,CAV2,FN1,MYLK,T  HBS1,PPP1CB,COL5A1,  COL4A2,LAMA5,COL5A2,  COL4A6,RAC2,PRKCG,  COL6A2,COL4A1,ERBB2,  LAMB1,ILK,CCND1,THBS3,  ITGA5 | ITGB6,BCL2,VCL,BRAF,  LAMA1,SRC,MET,GSK3B,FLNC,JUN, HRAS,CRKL,PAK2 |
| Wnt signaling pathway (04310) | 8.73 | 36/149 | WNT16,WNT4,WNT2,NKD2,  CTNNB1,MAPK10,FZD1,  PLCB1,SMAD3,CTNNBIP1,  WNT5A,PPP2R5B,RAC2,  WNT2B,FRAT1,PPP2R1B,  PRKCG,PPP2R5A,CSNK1E,  CCND1,NFATC4,DAAM2, LRP6 | PPP2CA,DAAM1,PPP3CC, NFATC1,FBXW11,GSK3B,  FZD6,JUN,APC2,WNT3,EP300,  CREBBP,CSNK2A1 |
| TGF-beta signaling pathway (04350) | 7.01 | 22/82 | BMP5,ID4,ACVRL1,RBL2,ID1,THBS1,SMAD3,ID3,BMPR1A,PPP2R1B,DCN,TFDP1,TGFB2,  THBS3 | ACVR1C,NOG,BMPR1B,  PPP2CA,EP300,CREBBP |
| Regulation of actin cytoskeleton (04810) | 6.37 | 42/207 | IQGAP2,DOCK1,BDKRB2,  ARHGEF6,PDGFRB,ITGB8,  PDGFRA,TMSB4Y,FGFR4,  ITGB5,FGF10,GSN,ITGA2, PDGFD,FN1,MYLK,FGFR2,  PPP1CB,F2R,MYH10,RAC2,  SSH2,ARPC1A, LIMK1,RRAS, SSH3,ITGA5 | ITGB6,CD14,VCL,SLC9A1,  BRAF,RDX,LIMK2,FGF13,  FGF2,EZR,APC2,HRAS,CRKL,  PIP5K1A,PAK2 |
| Pathways in cancer (05200) | 6.28 | 62/327 | WNT16,WNT4,WNT2,JUP,  CTNNB1,PDGFRB,HGF,  MAPK10,PDGFRA,FZD1,  LAMA2,FGF10,ITGA2,FN1, FGFR2,SMAD3,COL4A2,  WNT5A,LAMA5,IKBKB,  COL4A6,RAC2,WNT2B,STK36,PRKCG,SUFU,COL4A1,PPARG,PLD1,ERBB2,LAMB1,CCND1,  TGFB1, STAT3,HDAC1, TGFB2 | ACVR1C,RUNX1,BCL2,CEBPA, BRAF,LAMA1,CDK6,MDM2,  PIAS4,HDAC2,FGF13,FGF2,  MET,ETS1,TCEB2,GSK3B,FZD6,JUN,APC2,WNT3,EP300,  CREBBP,HRAS,TRAF6,CBL,  CRKL |
| Axon guidance(04360) | 6.22 | 29/128 | CXCL12,DPYSL2,SEMA5A,  EPHA7,SLIT3,EPHB6,ABLIM3,EPHA3,SEMA6D,SEMA4B,  NRP1,RAC2,SEMA3A,UNC5C,ROBO1,LIMK1,NFATC4 | EPHA2,NTN4,GNAI1,LIMK2, PPP3CC,MET,NFATC1,GSK3B, RHOD,SRGAP1,HRAS,PAK2 |
| Cell cycle (04110) | 5.96 | 27/112 | CDC25B,ANAPC5,CDKN2C,  RBL2,CDC16,SMAD3,ANAPC7,CCNB1,CCNB2,CCND1,TFDP1,TGFB1,HDAC1 | GADD45B,GADD45A,CDK6,  MDM,HDAC2,TGFB2,CDC14B, ANAPC11,GSK3B,ORC4L,EP300,CREBBP,ORC5L,ANAPC1 |
| Phosphatidylinositol signaling system (04070) | 5.30 | 1/6 | PLCB1,SYNJ2,PRKCG | DGKI,DGKH,PIP5K1A |
| p53 signaling pathway (04115) | 5.12 | 17/68 | THBS1,CCNG2,CCNB1,CCNB2, CCND1 | GADD45B,PPM1D,TNFRSF10B, GADD45A,BBC3,CDK6,MDM2, LRDD,PMAIP1,RCHY1,MDM4, SESN2 |
| Notch signaling pathway (04330) | 4.94 | 13/47 | DLL1,JAG1,MAML3,MAML2,  NCSTN, HDAC1,DTX4 | NOTCH1,NOTCH2,HDAC2,  EP300,CREBBP,DLL3 |
| Prostate cancer (05215) | 4.87 | 19/89 | CTNNB1,PDGFRB,PDGFRA,  PDGFD,FGFR2,IKBKB,SRD5A1,CREB3L2,CREB3L1,ERBB2,  CCND1 | BCL2,CREB3,BRAF,MDM2,  GSK3B,EP300,CREBBP,HRAS |
| Melanogenesis (04916) | 4.79 | 20/101 | WNT16,WNT4,WNT2,CTNNB1,FZD1,PLCB1,WNT5A,WNT2B,PRKCG, CREB3L2,CREB3L1 | TYRP1,CREB3,GNAI1,GSK3B, FZD6,WNT3,EP300,CREBBP, HRAS |

The 1.5 fold change p<0.05 list of probe changes was used to determine the most significant KEGG pathways changing in stroma in the presence of BPH-1 spheroids. Pathways are ranked according to impact factor and the number of genes changing in each pathway shown. These are divided into up-regulated and down-regulated genes and the fold change shown.
