# Supplementary material for: Stroma Regulates Increased Epithelial Lateral Cell Adhesion in 3D Culture: A Role for Actin/Cadherin Dynamics
Source: PLoS One. 2011 Apr 18;6(4):e18796. doi: 10.1371/journal.pone.0018796 (PMC3078910; doi:10.1371/journal.pone.0018796)
Supplement: Table S4 — Primary antibodies and dilutions for immunofluorescence and Western Blotting. (DOC) [file pone.0018796.s008.doc]

**Supplementary Table S4: Primary antibodies and dilutions for immunoflourescence and Western Blotting**

| Antigen | Supplier | Dilution for tissue stains IHC | Dilution for 3D acini IHC | Dilution for WB |
| --- | --- | --- | --- | --- |
| E-Cadherin | R&D systems | 1:50 | 1:50 | 1:1000 |
| Dsg 2 IgM (33-3D) | D Garrod (Manchester University) | 1:50 | 1:50 | 1:1000 |
| Dsg 3 IgG (32-2B) | D Garrod (Manchester University) | 1:50 | 1:50 | N/A |
| Β actin (phallodin) | Sigma | N/A | 2 μg/ml | 1:10 000 |
| FGFR2 | Abcam | N/A | 1:50 | N/A |
| Phosphorylated Smad 2/3 | Santa Cruz | N/A | 1:200 | N/A |
| CXCR4 | Abcam | N/A | 1:200 | N/A |
