# Supplementary material for: Stroma Regulates Increased Epithelial Lateral Cell Adhesion in 3D Culture: A Role for Actin/Cadherin Dynamics
Source: PLoS One. 2011 Apr 18;6(4):e18796. doi: 10.1371/journal.pone.0018796 (PMC3078910; doi:10.1371/journal.pone.0018796)
Supplement: Table S5 — Secondary antibodies and dilutions for immunofluorescence and Western blotting. (DOC) [file pone.0018796.s009.doc]

**Supplementary Table S5: Secondary antibodies and dilutions for immunoflourescence and Western blotting**

|  | Supplier | Dilution for tissue stains IHC | Dilution for 3D acini IHC | Dilution for WB |
| --- | --- | --- | --- | --- |
| Goat anti-mouse alexa 568 (IgG and IgM) | Molecular probes | 1:200 | 1:500 | N/A |
| Goat anti-mouse alexa 488 | Molecular probes | 1:200 | 1:500 | N/A |
| Goat anti- mouse Cy3 | GE Healthcare | N/A | N/A | 1:1250 |
| Goat anti-mouse Cy5 | GE Healthcare | N/A | N/A | 1:1250 |
| Goat anti- mouse HRP | Dako | N/A | N/A | 1:5000 |
| Rabbit anti-goat 488 | Molecular probes | N/A | 1:500 | N/A |
