# Supplementary material for: Stroma Regulates Increased Epithelial Lateral Cell Adhesion in 3D Culture: A Role for Actin/Cadherin Dynamics
Source: PLoS One. 2011 Apr 18;6(4):e18796. doi: 10.1371/journal.pone.0018796 (PMC3078910; doi:10.1371/journal.pone.0018796)
Supplement: Table S6 — Primers for RT-PCR. (DOC) [file pone.0018796.s010.doc]

**Supplementary Table S6: Primers for RT-PCR**

| Gene | Forward | Reverse |
| --- | --- | --- |
| Dsg1 | 5'-GGTGGGGCAGATGGCATGTCAGC-3' | 5'-TCCCACTTTATCATTTGATCCC-3' |
| Dsg2 | 5'-ACCACCTGAAGACAAGGTGG-3' | 5'-TGGTTTCAGTGGTCATGATAGC-3' |
| Dsg3 | 5'-GATAGTGACAGAAAGGGTGA-3' | 5'-CTACAATTTGAATAGTACTTG-3' |
| E-Cadherin | 5'-TTTGTACAGATGGGGTCTTGC-3' | 5'-CAAGCCCACTTTTCATAGTTCC-3' |
| GAPDH | 5'-AAGGTGAAGGTCGGAGTCAA-3' | 5'-GGACACGGAAGGCCATGCCA-3' |
