# Supplementary material for: Stroma Regulates Increased Epithelial Lateral Cell Adhesion in 3D Culture: A Role for Actin/Cadherin Dynamics
Source: PLoS One. 2011 Apr 18;6(4):e18796. doi: 10.1371/journal.pone.0018796 (PMC3078910; doi:10.1371/journal.pone.0018796)
Supplement: Table S7 — Primers and probes for real time RT-PCR. (DOC) [file pone.0018796.s011.doc]

**Supplementary Table S7: Primers and probes for real time RT-PCR**

|  | Forward | Reverse | Probe |
| --- | --- | --- | --- |
| E-Cad | 5'-GAAAATCTGAAAGCGGCTGATAC-3' | 5'-CGGAACCGCTTCCTTCATAG-3' | 5'-ACCCCACAGCCCCGCCTTATGA-3' |
| Dsg2 | 5'-GGGACCCGCAGCAATTG-3' | 5'-CACATCAGCAGTAAAAGTGGTACCA-3' | 5'-CTCATGATTTTGGCCTTTCTGCTCCTGC-3' |
| Dsg3 | 5'-CAGTGTTCCTGGCAACCTAGCT-3' | 5'-ATATTAGACGGGAGCAAGGATCCT-3' | 5'-CCCAACGCAGCTACGAGGGTCACAT-3' |
| Pak1 | 5’-GGGCCCAAGGTTGACATCT-3’ | 5’-GGAGGCTCCCCTTCAATCAT-3’ | 5’-CCTGGGCATCATGGCCATCGA-3’ |
| GAPDH | 5'-CCAGGTGGTCTCCTCTGACTTC-3' | 5'-GTGGTCGTTGAGGGCAATG-3' | 5'- ACAGCGACACCCACTCCTCCACCTT-3' |
